# Enhanced predictive validity of integrative models for refractory hyperthyroidism considering baseline and early therapy characteristics: a prospective cohort study

**DOI:** 10.1186/s12967-024-05129-3

**Published:** 2024-03-29

**Authors:** Xinpan Wang, Tiantian Li, Yue Li, Qiuyi Wang, Yun Cai, Zhixiao Wang, Yun Shi, Tao Yang, Xuqin Zheng

**Affiliations:** Department of Endocrinology, The First Affiliated Hospital of Nanjing Medical University, No.300, Guangzhou Road, Gulou District, Nanjing, Jiangsu China

**Keywords:** Graves’ disease, Refractory, Predictive model, Baseline characteristics, Early therapy, Drug dosage

## Abstract

**Background:**

A subset of Graves’ disease (GD) patients develops refractory hyperthyroidism, posing challenges in treatment decisions. The predictive value of baseline characteristics and early therapy indicators in identifying high risk individuals is an area worth exploration.

**Methods:**

A prospective cohort study (2018–2022) involved 597 newly diagnosed adult GD patients undergoing methimazole (MMI) treatment. Baseline characteristics and 3-month therapy parameters were utilized to develop predictive models for refractory GD, considering antithyroid drug (ATD) dosage regimens.

**Results:**

Among 346 patients analyzed, 49.7% developed ATD-refractory GD, marked by recurrence and sustained Thyrotropin Receptor Antibody (TRAb) positivity. Key baseline factors, including younger age, Graves’ ophthalmopathy (GO), larger goiter size, and higher initial free triiodothyronine (fT3), free thyroxine (fT4), and TRAb levels, were all significantly associated with an increased risk of refractory GD, forming the baseline predictive model (Model A). Subsequent analysis based on MMI cumulative dosage at 3 months resulted in two subgroups: a high cumulative dosage group (average ≥ 20 mg/day) and a medium–low cumulative dosage group (average < 20 mg/day). Absolute values, percentage changes, and cumulative values of thyroid function and autoantibodies at 3 months were analyzed. Two combined predictive models, Model B (high cumulative dosage) and Model C (medium–low cumulative dosage), were developed based on stepwise regression and multivariate analysis, incorporating additional 3-month parameters beyond the baseline. In both groups, these combined models outperformed the baseline model in terms of discriminative ability (measured by *AUC*), concordance with actual outcomes (66.2% comprehensive improvement), and risk classification accuracy (especially for Class I and II patients with baseline predictive risk < 71%). The reliability of the above models was confirmed through additional analysis using random forests. This study also explored ATD dosage regimens, revealing differences in refractory outcomes between predicted risk groups. However, adjusting MMI dosage after early risk assessment did not conclusively improve the prognosis of refractory GD.

**Conclusion:**

Integrating baseline and early therapy characteristics enhances the predictive capability for refractory GD outcomes. The study provides valuable insights into refining risk assessment and guiding personalized treatment decisions for GD patients.

**Supplementary Information:**

The online version contains supplementary material available at 10.1186/s12967-024-05129-3.

## Background

Hyperthyroidism is characterized by excessive circulation of thyroid hormones, resulting from increased synthesis and secretion or release of stored thyroid hormones. Graves' disease (GD), an autoimmune form of hyperthyroidism, accounts for 60–80% of cases of thyrotoxicosis [1, 2]. The main treatments of GD include: antithyroid drugs (ATD), radioactive iodine (RAI), and thyroid surgery. Methimazole (MMI) is often the primary choice among antithyroid drugs due to its relatively long half-life, high efficacy and relatively minor side effects [3]. The latest hyperthyroidism guidelines from the American Thyroid Association (2016) and the European Thyroid Association (2018) recommend maintaining ATD treatment for approximately 12–18 months. ATD can be withdrawn when thyroid-stimulating hormone (TSH) and thyrotropin receptor antibody (TRAb) levels normalize [4, 5]. Patients are considered to be in remission if they have normal serum TSH, free thyroxine (fT4), and total triiodothyronine (T3) levels for 1 year after ATD withdrawal. The recurrence rates among GD patients—ranging from 30 to 70%—vary significantly across different countries or regions [4, 6]. Compared to patients with normal TRAb, those with high TRAb at the end of ATD therapy have a significantly higher recurrence rate. Some studies report that the levels of TRAb in some patients remain high even after more than 2 years of treatment, which disqualifies them from treatment discontinuation [7–9]. GD patients with persistent hyperthyroidism who do not respond to ATD therapy or are prone to relapse after remission are considered as refractory GD [10].

In clinical practice, there is no universally agreed definition of refractory GD. Some scholars define refractory GD as a condition characterized by severe complications such as liver damage, blood cell reduction, GD related heart disease or vasculitis [11, 12]. Others believe that refractory GD refers to the presence of resistance or insensitivity to both ATDs and beta-blockers, where the hyperthyroid state cannot be normalized [13–15]. Alternatively, it may be considered when hyperthyroid symptoms disappear after several months of standardized drug therapy, but the biochemical hyperthyroid state persists with elevated fT4 and reduced TSH [16–18]. Additionally, patients with suboptimal response to a single RAI or surgery, requiring repeated treatments can also be classified as refractory GD patients [19]. This study attempted to use a composite endpoint outcome to describe refractory GD: (a) Failure to achieve withdrawal criteria after a course of standardized ATD therapy, especially with persistent positive TRAb; (b) Meeting withdrawal criteria and entering a remission phase but experiencing a recurrence of biochemical hyperthyroidism within a short period. In this study, the maximum therapy duration was restricted to two years, with a post-withdrawal observation period of one year. Therefore, we defined “refractory GD” as the hyperthyroidism condition unable to achieve withdrawal criteria after 2 years of ATD treatment or the recurrence of biochemical hyperthyroidism within one year after reaching the withdrawal criteria.

Various factors, mostly based on clinical characteristics and laboratory data at baseline—including age, gender, smoking history, goiter size, and thyroid hormone levels at initial diagnosis—have been examined for their predictive value for refractory GD [20–23]. However, because patients with similar baseline characteristics often differ in their drug responsiveness and hormonal changes during ATD therapy, their overall treatment outcomes and prognosis cannot be easily predicted based on these baseline characteristics [24–26]. Therefore, effective refractory risk factors must be further investigated, which the present study attempts to do. This study carefully analyzed individual characteristics and early therapy indicators, built three risk prediction models, and evaluated their predictive validity for refractory GD. Integrating baseline and early therapy characteristics enhances the predictive capability for refractory GD outcomes. This research could assist healthcare professionals and patients in making proper treatment decisions.

## Methods

### Participants

Between 2018 and 2022, 597 newly diagnosed adult patients with GD were screened at the First Affiliated Hospital of Nanjing Medical University. We excluded 251 patients due to the following reasons: 66 were undergoing active treatment and had not finished two years of treatment; 23 had a follow-up period of less than 1 year after withdrawal; 12 switched to RAI; 9 developed thyroid malignant tumors during therapy; 7 became pregnant during the treatment; 24 used medications beyond the prescribed guidelines, such as switched to propylthiouracil (PTU) for GD or used high-dose steroids for Graves’ Ophthalmopathy (GO); 78 had irregular follow-up or course of therapy; and 32 were lost to follow-up. Finally, 346 patients were included in this study.

The diagnosis of GD was based on the established criteria, including clinical features, decreased TSH levels (< 0.270 mIU/L), elevated fT4 (> 22.0 pmol/L), positive TRAb (> 1.5 IU/L), radioactive iodine uptake, or thyroid ultra-sound with Doppler [27]. The mandatory and supporting diagnostic criteria included the former and latter three items, respectively. All included patients in this study had positive TRAb. Exclusion criteria included: taking medications that could affect thyroid efficacy within the three months before enrollment, a history of thyroid surgery, other thyroid diseases such as hyperfunctioning adenomas or subacute thyroiditis, other autoimmune diseases or malignancies, and pregnancy or lactation.

### Therapy criteria

All participants in the study were treated with methimazole (Merck, Germany) for hyperthyroidism. The dosage of MMI ranged from 10 to 30 mg/day initially, with a maintenance dose of 2.5–10 mg/day in most cases. Levothyroxine was allowed for thyroid hormone supplementation in case of drug-induced hypothyroidism. Additional medications—such as beta-blockers, B-complex vitamins, and drugs to elevate white blood cell count—were permitted. Drugs affecting MMI efficacy and observation indicators—such as PTU, iodine-containing medicines, corticosteroids (intravenous or oral)—were not permitted.

The treatment plan involved individualized adjustments by attending physicians, following either titration or block-replacement protocols [28], but not by a randomized design. Regular reminders for follow-up visits were conducted through phone calls and online consultations. Patients were generally required to follow up offline. The data—including thyroid function, thyroid autoantibodies, and medication dosage—were recorded on a standardized paper form during each follow-up visit. Assessments were conducted monthly for the first 6 months and every 2 months thereafter until the withdrawal criteria were met, including maintaining thyroid function within the normal range or mild drug-induced hypothyroidism after approximately 12 to 18 months of regular MMI therapy while TRAb was negative. Patients with persistent TRAb positivity after 2 years were advised by physicians, considering patient preferences, to either extend the treatment period or attempt withdrawal. Refractory hyperthyroidism was defined as recurrence within 1 year after withdrawal from therapy for up to 2 years or persistent TRAb positivity after more than 2 years of regular follow-up. Patient information, including age, gender, smoking history, family history, and clinical parameters, was recorded. This study received approval from the Ethics Review Committee of the First Affiliated Hospital of Nanjing Medical University, and all patients provided written informed consent.

### Laboratory measurement

Serum levels of fT3, fT4, TSH, thyroid peroxidase antibody (TPOAb), thyroglobulin antibody (TgAb), and TRAb were measured with MODULAR ANALYTICS E170 fully automated electrochemiluminescence immunoassay system and matching reagent kits (Roche Diagnostics, Germany). Normal reference ranges were as follows: fT3 3.10–6.80 pmol/L, fT4 12.00–22.00 pmol/L, TSH 0.270–4.200 mIU/L, TPOAb < 34.0 IU/mL, TgAb < 115.0 IU/mL, TRAb 0.0–1.5 IU/L.

### Thyroid volume measurement

Thyroid ultrasound examinations were performed on participants using the Siemens color Doppler ultrasound diagnostic instrument (Germany) with a probe frequency of 5–15 Hz. Measurements include the length (a), height (b), and thickness (c) of both the left and right thyroid lobes in millimeters. The formula for calculating thyroid volume is as follows: Left lobe 0.479 × (a × b × c)/1000 + Right lobe 0.479 × (a × b × c)/1000[29].

## Statistical analysis

R-4.3.0 and SPSS 27.0 were used for statistical analysis, Python 3.9 for curve fitting, and Graphpad Prism 9.0 for plotting. Continuous variables were presented as mean ± standard deviation if normally distributed; otherwise, medians and interquartile ranges were used. Normal distribution was assessed using t-tests or ANOVA for continuous variables, and non-parametric tests for non-normally distributed ones. Categorical variables were analyzed using the chi-square test or Fisher's exact test. The rank sum test was used for hierarchical data. Multiple imputations were done with R using five iterations, including all predictor and outcome variables. The receiver operating characteristic (*ROC*) curve determined optimal cutoff values for continuous variables. Thyroid function changes were modeled with polynomial fits, and cumulative values were calculated. All 346 data were used for analysis, with bootstrap resampling for internal validation. There was no external validation in this study.

Hyperthyroidism refractoriness was the dependent variable. All baseline data were used as independent variables for univariate logistic regression analyses. Variables with *P* < 0.1 in the univariate analysis were chosen for the further multivariate logistic regression analysis. Those with *P* < 0.05 were considered as baseline model parameters for refractory GD, leading to the development of the baseline predictive model (Model A). Meanwhile, absolute values, cumulative values, and percentage changes in thyroid function and autoantibody levels at three months of therapy were used as independent variables for stepwise regression analysis. Model parameters of the 3-month therapy in the high cumulative MMI dosage group and the medium–low cumulative MMI dosage group were selected separately according to the results of stepwise regression analysis. Multivariate logistic regression analyses were performed based on all parameters from Model A and the selected parameters of the 3-month therapy. This resulted in the development of early-stage combined predictive models for the high cumulative (Model B) and medium-to-low cumulative (Model C) MMI dosage groups. A *P* < 0.05 was considered significant unless otherwise specified. Three multivariate models were developed in total: a baseline model for all newly diagnosed GD patients, an early treatment model for patients with high cumulative MMI dosage, and an early treatment model for patients with medium-to-low cumulative MMI dosage. Models were presented as nomogram plots. *ROC* curves assessed discriminative ability. Calibration curves, the Hosmer–Lemeshow (*HL*) test, and mean absolute error (*MAE*) evaluated accuracy. Models were compared based on area under the curve (*AUC*), consistency of outcome, and risk classification.

In addition, the random forest algorithm in machine learning was applied to create three sensitivity analysis validation models, using hyperthyroidism refractoriness as the dependent variable. All potential independent variables were converted to categorical variables. Mean decrease Gini (*MDG*) determined variable importance. Considering baseline data of all members in the analysis cohort, Model A + was established based on the *MDG* ranking. For absolute values, cumulative values, and percentage changes in thyroid function and autoantibody levels at three months of treatment, similar independent variable selection was conducted. Parameters for the high cumulative and medium–low cumulative MMI dosage groups during the 3-month treatment were chosen based on the *MDG* rankings. Subsequently, utilizing all parameters from Model A + and the selected 3-month treatment parameters, two random forest models were established: Model B + (high cumulative MMI dose group) and Model C + (medium–low cumulative MMI dose group). Among the random forest models, discriminative ability was assessed using *ROC* curves, and accuracy was evaluated with *MAE*. Model comparisons were conducted through *AUC*.

## Results

### Baseline characteristics

**Fig. 1:**
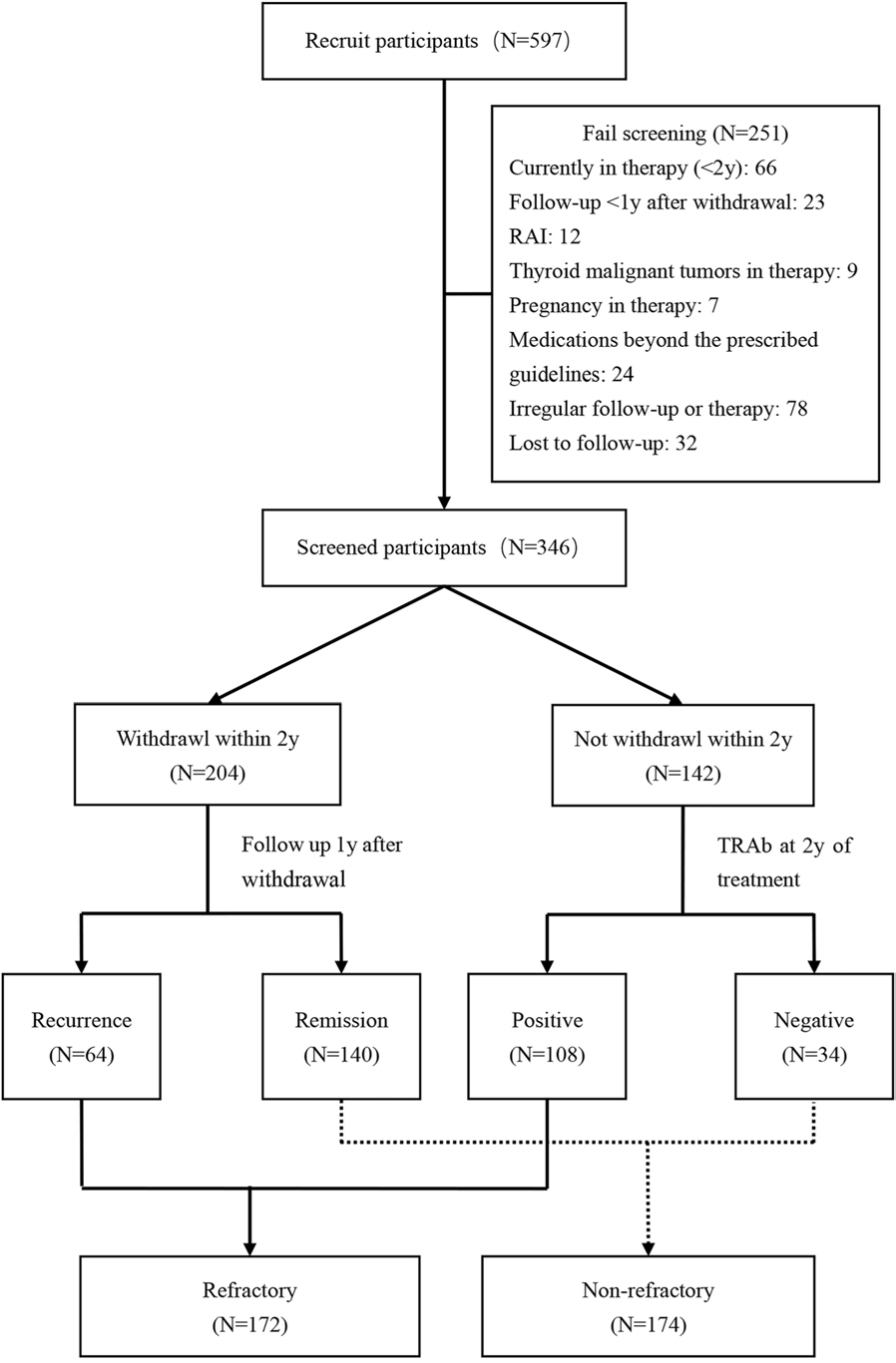
Flowchart of screening and composition for patient with Graves’ disease. *y* year, *RAI* radioactive iodine, *TRAb* thyrotropin receptor antibody

Out of the initial 597 newly diagnosed GD patients screened for this prospective study, 251 individuals were excluded. This resulted in a final cohort of 346 GD patients for the analysis and model development. Within the final cohort, 49.7% (172/346) of the patients ultimately developed refractory hyperthyroidism. Among these patients, 37.2% (64/172) experienced recurrence within 1 year after treatment withdrawal, while 62.8% (108/172) remained TRAb-positive after 2-year therapy (Fig. 1).

Baseline characteristics are presented in Table 1. Compared to non-refractory patients, refractory patients were younger by 7 years (*P* < 0.001), had a higher prevalence of GO (*P* < 0.001), larger goiter size (*P* < 0.001), higher serum levels of fT3 (*P* = 0.008), fT4 (*P* = 0.021) and TRAb (*P* < 0.001) at the initial diagnosis. No differences were observed between the two groups concerning gender, smoking behavior, family history, initial TSH, initial TPOAb, and initial TgAb before therapy.

**Table 1:** Baseline characteristics of the patients in the refractory and non-refractory groups

Baseline characteristics	Refractory (n = 172)	Non-refractory (n = 174)	*P* value
Age, year	31 (26–42)	38 (28–47)	< 0.001
Female sex, n (%)	131 (76.2%)	135 (77.6%)	0.754
Current smoking, n (%)	22 (12.8%)	14 (8.05%)	0.148
GD family history, n (%)	18 (10.3%)	18 (10.5%)	0.971
GO, n (%)	56 (32.6%)	29 (16.7%)	< 0.001
Initial goiter size (cm^3^)	20.5 (15.1–29.4)	16.7 (11.0–23.3)	< 0.001
Initial fT3 (pmol/L)	27.6 (15.3–40.1)	22.5 (15.5–33.0)	0.008
Initial fT4 (pmol/L)	70.3 (44.3–100.0)	57.5 (41.9–90.4)	0.021
Initial TSH (mIU/L)	0.005 (0.005–0.005)	0.005 (0.005–0.005)	0.564
Initial TPOAb (IU/mL)	166.4 (22.9–352.4)	100.1 (18.6–289.2)	0.128
Initial TgAb (IU/mL)	199.1 (26.4–497.6)	232.4 (32.5–493.3)	0.767
Initial TRAb (IU/L)	15.1 (5.4–29.0)	7.5 (3.9–15.2)	< 0.001

*GO* Graves’ orbitopathy, *GD* Graves’ disease, *fT3* free triiodothyronine, *fT4* free thyroxine, *TSH* thyroid stimulating hormone, *TPOAb* thyroid peroxidase autoantibody, *TgAb* thyroglobulin autoantibody, *TRAb* thyroid stimulating hormone receptor autoantibody

### Baseline prediction model before therapy (Model A)

**Fig. 2:**
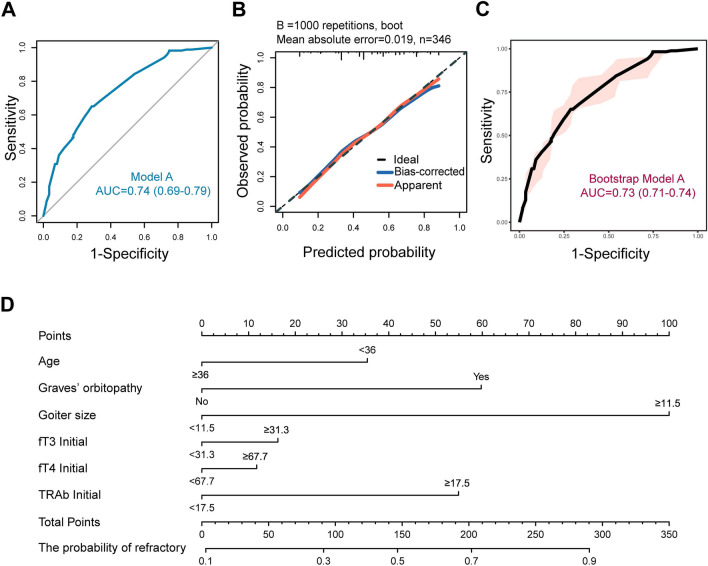
Visual analysis results of Model A. **A** *ROC* curve of analysis cohort. **B** Calibration plots of analysis and validation cohorts. **C** *ROC* curve of validation cohort. **D** Nomogram plot of analysis cohort. *ROC* receiver operating characteristic curve, *AUC* area under the curve, *fT3* free triiodothyronine, *fT4* free thyroxine, *TRAb* thyroid stimulating hormone receptor autoantibody. The nomogram plot is used by entering the categorical status of each patient-related factor, calculating scores for each item, and summing the total score to assess the risk of refractory GD

As shown in Table 2, univariate analysis revealed that, before the initiation of therapy, lower age (< 36 years), GO, larger goiter size (≥ 11.5 cm^3^), higher initial fT3 (≥ 31.3 pmol/L), fT4 (≥ 67.7 pmol/L) and TRAb (≥ 17.5 IU/L) levels were all associated with refractory GD. Smoking behavior, initial TPOAb, and initial TgAb were not associated with refractory GD. Multivariate analysis further indicated that lower age (*OR* = 1.7, *P* = 0.024), GO (*OR* = 2.5, *P* = 0.002), larger goiter size (*OR* = 4.6, *P* < 0.001), and higher TRAb (*OR* = 2.3, *P* = 0.001) were significantly associated with an increased odds ratio (*OR*) for refractory hyperthyroidism. Based on the variables selected from multivariate analysis (*P* < 0.05), we constructed a baseline predictive model for refractory GD, called Model A. The *ROC* curve (*AUC* = 0.74) and calibration plot (*HL* test *P* = 0.964) demonstrated good discriminative ability and calibration for this baseline model (Analysis cohort) (Fig. 2A and B). The validation cohort showed similar results (Fig. 2B and C). The baseline model was visualized with a nomogram plot (Fig. 2D).

**Table 2:** Refractory odds ratios for selected baseline characteristics in univariable and multivariable analyses

Baseline characteristics	Univariate analyses			Multivariate analyses^a^	
	Refractory, % (n/N)	*OR* (95% CI)	*P* value	*OR* (95% CI)	*P* value
Age, year					
≥ 36	39.4 (65/165)	Reference		Reference	
< 36	59.1 (107/181)	2.2 (1.4–3.4)	< 0.001	1.7 (1.1–2.7)	0.024
Current smoking, n (%)					
Yes	61.1 (22/36)	1.7 (0.8–3.4)	0.152		
No	48.4 (150/310)	Reference			
GO, n (%)					
Yes	65.9 (56/85)	2.4 (1.4–4.0)	0.001	2.5 (1.4–4.5)	0.002
No	44.4(116/261)	Reference		Reference	
Initial goiter size (cm^3^)					
≥ 11.5	56.5 (160/283)	5.5 (2.8–10.8)	< 0.001	4.6 (2.2–9.5)	< 0.001
< 11.5	19.0 (12/63)	Reference		Reference	
Initial fT3 (pmol/L)					
≥ 31.3	62.3 (76/122)	2.2 (1.4–3.5)	0.001	1.3 (0.6–2.7)	0.521
< 31.3	42.9 (96/224)	Reference		Reference	
Initial fT4 (pmol/L)					
≥ 67.7	59.6 (90/151)	2.0 (1.3–3.1)	0.001	1.2 (0.6–2.5)	0.629
< 67.7	42.1 (82/195)	Reference		Reference	
Initial TPOAb (IU/mL)					
≥ 67.7	53.1 (111/209)	1.4 (0.9–2.2)	0.119		
< 67.7	44.5 (61/137)	Reference			
Initial TgAb (IU/mL)					
≥ 660.0	58.6 (34/58)	1.5 (0.9–2.7)	0.139		
< 660.0	47.9 (138/288)	Reference			
Initial TRAb (IU/L)					
≥ 17.5	68.6 (81/118)	3.3 (2.1–5.3)	< 0.001	2.3 (1.4–3.9)	0.001
< 17.5	39.9 (91/228)	Reference		Reference	

*GO* Graves’ orbitopathy, *fT3* free triiodothyronine, *fT4* free thyroxine, *TSH* thyroid stimulating hormone, *TPOAb* thyroid peroxidase autoantibody, *TgAb* thyroglobulin autoantibody, *TRAb* thyroid stimulating hormone receptor autoantibody, *OR* odds ratio, *CI* confidence interval

^a^Multivariate analysis includes all characteristics with *P* < 0.1 in univariate analysis

### Cumulative MMI dosage analysis at 3 months of therapy

Thyroid function and thyroid autoantibody levels at 3 months of therapy differed between patients with refractory and non-refractory GD (Table 3). Refractory patients exhibited higher levels of fT3, fT4, TSH, TPOAb, TgAb, and TRAb, with greater percentage decreases in TgAb and TRAb levels. Additionally, cumulative values for TPOAb and TRAb were higher in refractory patients compared to non-refractory patients.

**Table 3:** Comparison of thyroid function and autoantibody indicators of the patients at 3 months of therapy

Characteristics of early therapy	Refractory (n = 172)	Non-refractory (n = 174)	*P* value
fT3 (3 m)^a^ (pmol/L)	6.7 (5.0–9.7)	6.2 (4.7–7.8)	0.037
fT3 (3 m percentage decrease)^b^ (%)	69.7 ± 16.6	70.4 ± 15.7	0.421
fT3 (3 m accumulation)^c^ (day*pmol/L)	1046.4 (823.4–1394.6)	998.1 (751.3–1299.1)	0.126
fT4 (3 m)^a^ (pmol/L)	20.7 (15.2–26.4)	18.6 (14.4–22.1)	0.023
fT4 (3 m percentage decrease)^b^ (%)	68.6 ± 17.8	66.7 ± 16.2	0.435
fT4 (3 m accumulation)^c^ (day*pmol/L)	2710.6 (2019.6–3539.5)	2770.3 (2077.2–3511.8)	0.957
TSH (3 m)^a^ (mIU/L)	1.5 (0.3–4.8)	1.9 (0.4–4.8)	0.093
TSH (3 m percentage increase)^b^ (%)	29,472.0 (4117.1–89,921.0)	36,701.0 (8772.7–102,865.7)	0.157
TSH (3 m accumulation)^c^ (day*mIU/L)	79.4 (7.3–242.2)	63.9 (5.4–208.4)	0.441
TPOAb (3 m)^a^ (IU/mL)	137.1 (39.0–312.5)	84.9 (25.6–201.8)	0.015
TPOAb (3 m percentage decrease)^b^ (%)	19.1 (2.6–33.8)	18.8 (11.4–36.0)	0.994
TPOAb (3 m accumulation)^c^ (day* IU/mL)	15,056.7 (3600.4–29,545.1)	8892.8 (1818.1–22,319.4)	0.009
TgAb (3 m)^a^ (IU/mL)	230.1 (48.1–442.9)	168.5 (19.5–436.7)	0.048
TgAb (3 m percentage decrease)^b^ (%)	38.1 (0.6–58.6)	21.7 (12.0–51.1)	0.003
TgAb (3 m accumulation)^c^ (day* IU/mL)	23,858.0 (4768.6–44,855.1)	19,148.7 (2914.0–45211.9)	0.512
TRAb (3 m)^a^ (IU/L)	6.5 (2.8–12.5)	4.7 (2.5–9.2)	0.045
TRAb (3 m percentage decrease)^b^ (%)	44.8 (25.6–63.2)	36.5 (56.5–17.1)	0.019
TRAb (3 m accumulation)^c^ (day* IU/L)	1081.9 (425.5–1634.8)	605.7 (316.7–1210.5)	< 0.001

*fT3* free triiodothyronine, *fT4* free thyroxine, *TSH* thyroid stimulating hormone, *TPOAb* thyroid peroxidase autoantibody, *TgAb* thyroglobulin autoantibody, *TRAb* thyroid stimulating hormone receptor autoantibody, *m* month

^a^Absolute serum levels at 3 months of MMI therapy

^b^Increase or decrease percentage of serum levels at 3 months of MMI therapy compared with the serum levels before therapy

^c^The area under the fitted curve of 3-month serum levels after the start of MMI therapy (the abscissa is days, the ordinate is the fT3/fT4/TSH/TPOAb/TgAb/TRAb level)

**Fig. 3:**
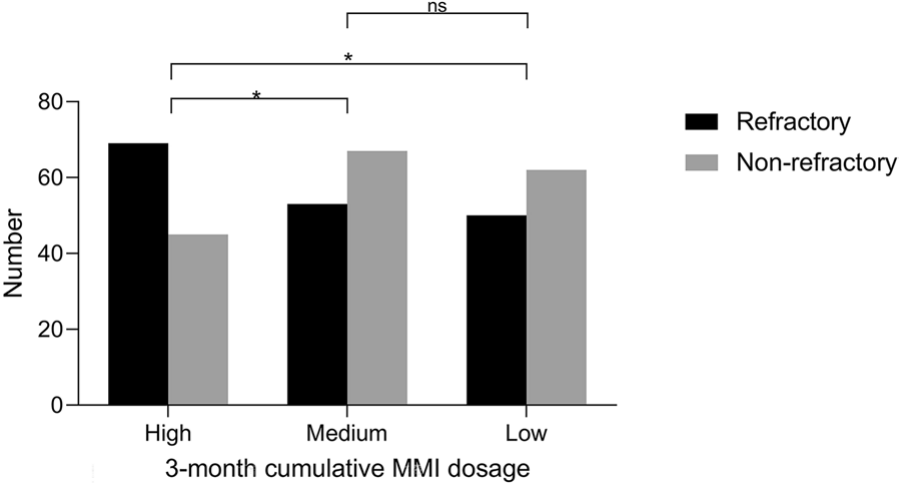
Distribution of patients by 3-month cumulative MMI dosage. *MMI* methimazole. *P < 0.05. High: 3-month cumulative MMI dosage ≥ 1730 mg. Medium: 3-month cumulative MMI dosage 1350–1730 mg, excludes 1730 mg. Low: 3-month cumulative MMI dosage < 1350 mg

To mitigate the confounding effects of antithyroid drugs on thyroid function and antibody changes, a subgroup analysis was conducted based on the cumulative dosage of MMI from 0 to 3 months (Fig. 3). Patients were categorized into high (≥ 1730 mg, N = 114), medium (1350–1730 mg, N = 120), and low (< 1350 mg, N = 112) cumulative dosage groups. Significant differences were found in the distribution of refractory GD among these three cumulative dosage groups (*P* = 0.017). Specifically, significant differences existed between the high and medium cumulative dosage groups (*P* = 0.013) and between the high and low cumulative dosage groups (*P* = 0.023), while no significant difference existed between the medium and low cumulative dosage groups (*P* > 0.05).

### Combined predictive model at 3 months of therapy (Models B and C)

Based on the subgroup analysis of cumulative MMI dosage mentioned above, the cohort was divided into a high cumulative MMI dosage group (≥ 1730 mg, average ≥ 20 mg/day, N = 114) and a medium–low cumulative MMI dosage group (< 1730 mg, average < 20 mg/day, N = 232) at 3 months of therapy. Thyroid function (fT3, fT4, TSH) and thyroid autoantibodies (TPOAb, TgAb, TRAb) were compared at the 3-month mark by analyzing the absolute values, percentage changes, and cumulative values.

**Fig. 4:**
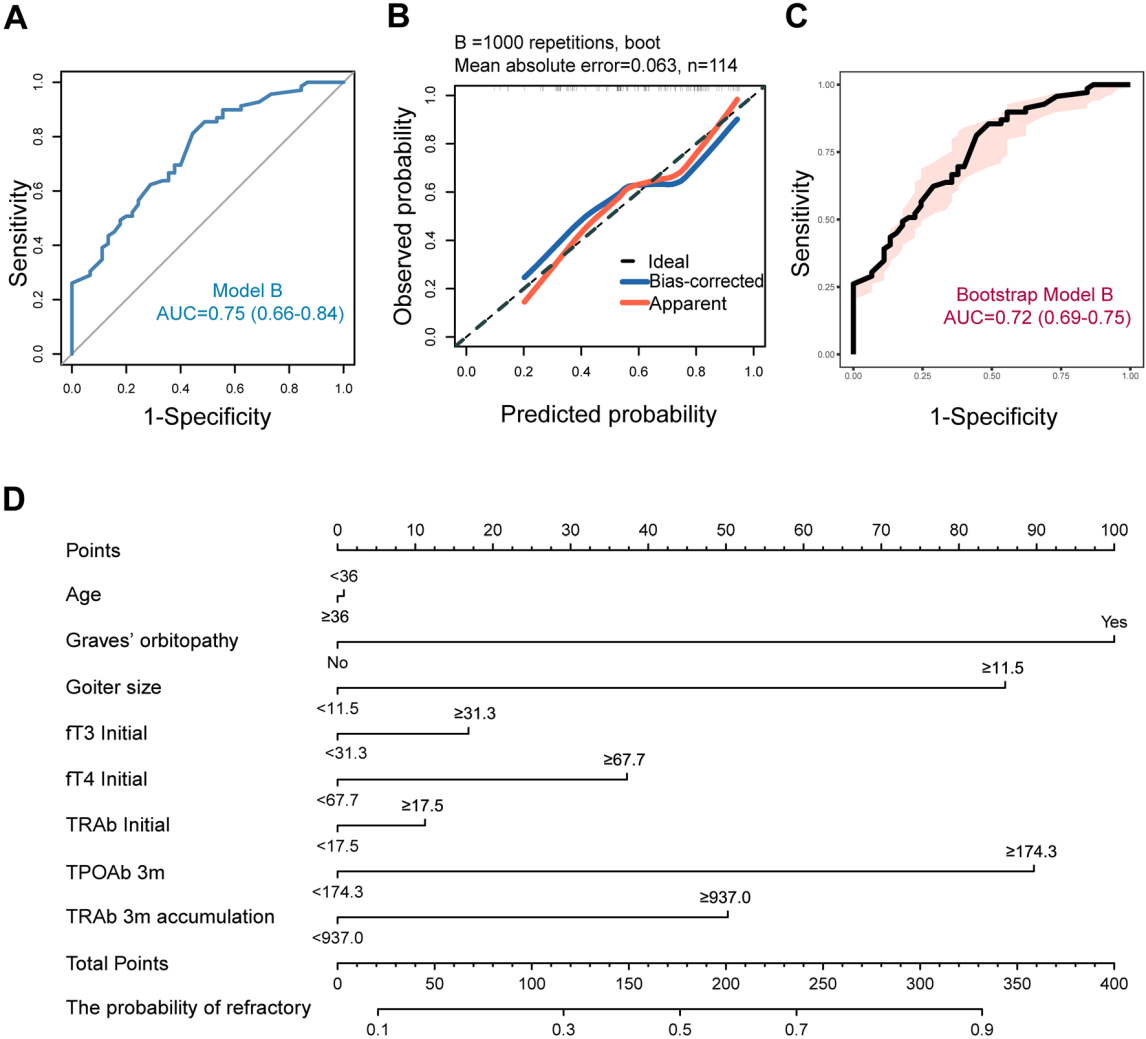
Visual analysis results of Model B. **A** *ROC* curve of analysis cohort. **B** Calibration plots of analysis and validation cohorts. **C** *ROC* curve of validation cohort. **D** Nomogram plot of analysis cohort. *ROC* receiver operating characteristic curve, *AUC* area under the curve, *fT3* free triiodothyronine, *fT4* free thyroxine, *TRAb* thyroid stimulating hormone receptor autoantibody, *TPOAb* thyroid peroxidase autoantibody, *m* month. The nomogram plot is used by entering the categorical status of each patient-related factor, calculating scores for each item, and summing the total score to assess the risk of refractory GD

For the high cumulative MMI dosage group, the univariate analysis identified that higher TPOAb and TgAb absolute values, a smaller percentage decrease in fT3, and higher cumulative values of TPOAb and TRAb were associated with refractory hyperthyroidism at 3 months. An additional table file shows this in more detail (see Additional file 1). To address multicollinearity, stepwise regression analysis was performed, resulting in the selection of two variables: absolute value of TPOAb at 3 months (*β* = 0.288, *VIF* = 1.004, *P* = 0.001) and cumulative TRAb at 3 months (*β* = 0.205, *VIF* = 1.004, *P* = 0.021). As shown in Table 4, age, GO, goiter, baseline fT3, baseline fT4, baseline TRAb, TPOAb absolute value at 3 months, and cumulative TRAb at 3 months were included in the combined predictive model (Model B) for the high cumulative MMI dosage group, incorporating clinical and laboratory data from baseline and the 3-month treatment point. The *ROC* (*AUC* = 0.75) and calibration curves (*HL* test *P* = 0.937) demonstrated good discriminative ability and calibration for Model B (Analysis cohort) (Fig. 4A and B). Similar results were observed in the validation cohort (Fig. 4B and C). The visualization of Model B is presented in a nomogram plot (Fig. 4D).

**Table 4:** Refractory odds ratios for characteristics of baseline and early therapy in high cumulative dosage subgroup

Characteristics of baseline and early therapy (High)^a^	Combined multivariate analyses		
	Refractory, % (n/N)	*OR* (95% CI)	*P* value
Age, year			
≥ 36	52.4 (22/42)	Reference	
< 36	65.3 (47/72)	1.0 (0.4–2.5)	0.980
GO, n (%)			
Yes	80.6 (25/31)	4.1 (1.3–13.4)	0.019
No	53.0 (44/83)	Reference	
Initial goiter size (cm^3^)			
≥ 11.5	63.2 (67/106)	3.4 (0.5–23.0)	0.216
< 11.5	25.0 (2/8)	Reference	
Initial fT3 (pmol/L)			
≥ 31.3	61.6 (45/73)	0.8 (0.2–2.8)	0.713
< 31.3	58.5 (24/41)	Reference	
Initial fT4 (pmol/L)			
≥ 67.7	60.2 (50/83)	1.7 (0.4–6.8)	0.457
< 67.7	61.3 (19/31)	Reference	
Initial TRAb (IU/L)			
≥ 17.5	68.5 (37/54)	1.2 (0.4–3.6)	0.783
< 17.5	53.3 (32/60)	Reference	
TPOAb (3 m)^b^ (IU/mL)			
≥ 174.3	76.9 (40/52)	3.5 (1.4–8.8)	0.006
< 174.3	46.8 (29/62)	Reference	
TRAb (3 m accumulation)^c^ (day* IU/L)			
≥ 937.0	70.3 (45/64)	2.0 (0.7–6.2)	0.209
< 937.0	48.0 (24/50)	Reference	

*GO*, Graves’ orbitopathy, *fT3* free triiodothyronine, *fT4* free thyroxine, *TPOAb* thyroid peroxidase autoantibody, *TRAb* thyroid stimulating hormone receptor autoantibody, *OR* odds ratio, *CI* confidence interval, *m* month

^a^3-month high cumulative MMI dosage group (≥ 1730 mg, average ≥ 20 mg/day, N = 114)

^b^Absolute serum TPOAb level at 3 months of MMI therapy

^c^The area under the fitted curve of 0–3 months serum TRAb level after the start of MMI therapy (the abscissa is days, the ordinate is the TRAb level)

**Fig. 5:**
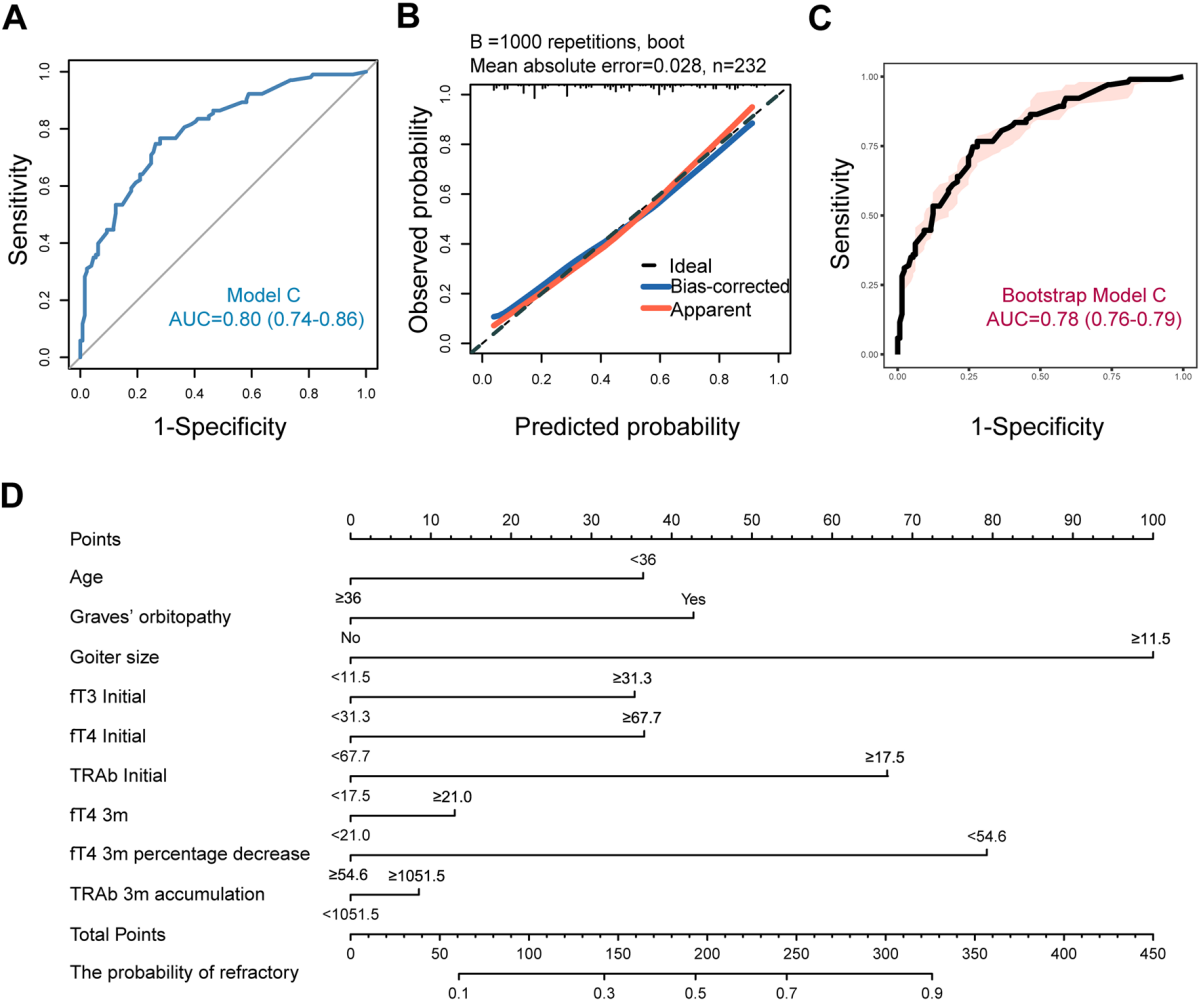
Visual analysis results of Model C. **A** *ROC* curve of analysis cohort. **B** Calibration plots of analysis and validation cohorts. **C** *ROC* curve of validation cohort. **D** Nomogram plot of analysis cohort. *ROC* receiver operating characteristic curve, *AUC* area under the curve, *fT3* free triiodothyronine, *fT4* free thyroxine, *TRAb* thyroid stimulating hormone receptor autoantibody, *m* month. The nomogram plot is used by entering the categorical status of each patient-related factor, calculating scores for each item, and summing the total score to assess the risk of refractory GD

For the medium–low cumulative MMI dosage group, univariate analysis revealed that higher absolute values of fT3, fT4, and TRAb; a smaller percentage decrease in fT4; a smaller percentage increase in TSH; and higher cumulative values of fT4 and TRAb were all associated with refractory hyperthyroidism at the 3-month mark. An additional table file shows this in more detail (see Additional file 2). Within the medium–low dosage group, stepwise regression analysis was employed to select variables related to thyroid function and autoantibodies among the 18 considered factors. Three variables were ultimately chosen: absolute value of fT4 at 3 months (*β* = 0.169, *VIF* = 1.031, *P* = 0.010), percentage decrease in fT4 at 3 months (*β* = − 0.133, *VIF* = 1.025, *P* = 0.048), and cumulative TRAb at 3 months (*β* = 0.257, *VIF* = 1.009, *P* < 0.001). As presented in Table 5, age, GO, goiter, baseline fT3, baseline fT4, baseline TRAb, fT4 absolute value at 3 months, percentage decrease in fT4 at 3 months, and cumulative TRAb at 3 months were incorporated into the combined predictive model (Model C) for the medium–low cumulative MMI dosage group. This model also encompassed clinical characteristics and laboratory data from the baseline and the 3-month therapy point. The *ROC* (*AUC* = 0.80) and calibration curves (*HL* test *P* = 0.699) demonstrated good discriminative ability and calibration for Model C (Analysis cohort) (Fig. 5A and B). Similar results were observed in the validation cohort (Fig. 5B and C). The visualization of Model C is presented in a nomogram plot (Fig. 5D).

**Table 5:** Refractory odds ratios for characteristics of baseline and early therapy in medium–low cumulative dosage subgroup

Characteristics of baseline and early therapy (Medium–low)^a^	Combined multivariate analyses		
	Refractory, % (n/N)	*OR* (95% CI)	*P* value
Age, year			
≥ 36	35.0 (43/123)	Reference	
< 36	55.0 (60/109)	1.8 (1.0–3.4)	0.054
GO, n (%)			
Yes	57.4 (31/54)	2.0 (1.0–4.2)	0.060
No	40.4 (72/178)	Reference	
Initial goiter size (cm^3^)			
≥ 11.5	52.5 (93/177)	5.2 (2.2–12.6)	< 0.001
< 11.5	18.2 (10/55)	Reference	
Initial fT3 (pmol/L)			
≥ 31.3	63.3 (31/49)	1.8 (0.6–5.3)	0.289
< 31.3	39.3 (72/183)	Reference	
Initial fT4 (pmol/L)			
≥ 67.7	58.8 (40/68)	1.8 (0.7–5.1)	0.244
< 67.7	38.4 (63/164)	Reference	
Initial TRAb (IU/L)			
≥ 17.5	68.8 (44/64)	3.0 (1.1–8.5)	0.036
< 17.5	35.1 (59/168)	Reference	
fT4 (3 m)^b^ (pmol/L)			
≥ 21.0	57.5 (50/87)	1.2 (0.6–2.4)	0.536
< 21.0	36.6 (53/145)	Reference	
fT4 (3 m percentage decrease)^c^ (%)			
≥ 54.6	39.5 (62/157)	Reference	
< 54.6	54.7 (41/75)	3.7 (1.7–7.9)	0.001
TRAb (3 m accumulation)^d^ (day* IU/L)			
≥ 1051.5	62.0 (49/79)	1.2 (0.4–3.0)	0.775
< 1051.5	35.3 (54/153)	Reference	

*GO* Graves’ orbitopathy, *fT3* free triiodothyronine, *fT4* free thyroxine, *TRAb* thyroid stimulating hormone receptor autoantibody, *OR* odds ratio, *CI* confidence interval; m, month

^a^3-month medium–low cumulative MMI dosage group (< 1730 mg, average < 20 mg/day, N = 232)

^b^Absolute serum fT4 level at 3 months of MMI therapy

^c^Decrease percentage of serum fT4 level at 3 months of MMI therapy compared with the serum levels before therapy

^d^The area under the fitted curve of 0–3 months serum TRAb level after the start of MMI therapy (the abscissa is the days, the ordinate is the TRAb level)

### Enhancing outcome prediction: impact of combined baseline and 3-month therapy characteristics

**Fig. 6:**
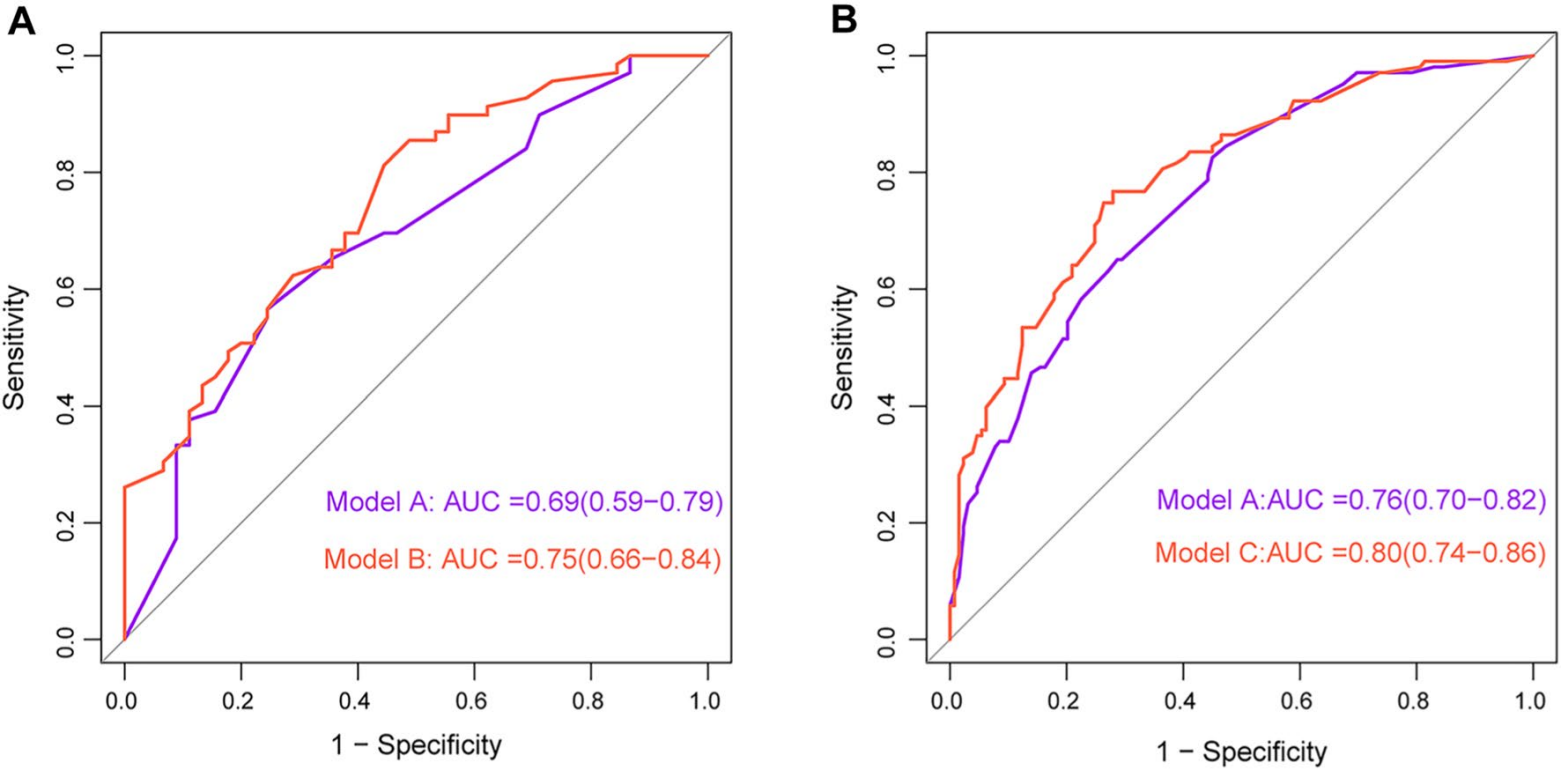
Comparison of *AUC* between different logistic regression models. **A** Model B vs. Model A. **B** Model C vs. Model A. Model A: baseline predictive model for total group (N = 346). Model B: combined model of high cumulative MMI dosage group (≥ 1730 mg, average ≥ 20 mg/day, N = 114) at 3 months of therapy. Model C: combined model of medium–low cumulative MMI dosage group (< 1730 mg, average < 20 mg/day, N = 232) at 3 months of therapy

Model A was built with baseline characteristics. Models B and C were developed by incorporating characteristics from both the baseline and the 3-month therapy period. Assessing the 3-month high cumulative MMI dosage group, Model B outperformed Model A with a higher *AUC* (0.75 vs. 0.69, *P* = 0.046) (Fig. 6A). Similarly, for the 3-month medium–low cumulative MMI dosage group, Model C exhibited a higher *AUC* than Model A (0.80 vs. 0.76, *P* = 0.020) (Fig. 6B). Whether in the high or medium–low MMI cumulative dosage group, the combined models were superior in distinguishing refractory GD outcomes compared to models relying solely on the baseline information.

The actual outcomes indicate an overall refractory risk of 49.7%. Compared to the baseline model (Model A), following reevaluation, Model B showed a risk increase and decrease in 51.8% (59/114) and 48.2% (55/114) of patients, respectively, and a risk change exceeding 20% in 29.0% (33/114) of patients. The risk predictions for 65.0% (74/114) of patients in Model B aligned more closely with the actual outcomes. Similarly, compared to Model A, Model C resulted in a risk increase in 48.2% (112/232) of patients and a decrease in 51.7% (120/232), with a risk change exceeding 20% in 7.3% (17/232) of patients. The risk predictions of 66.8% (155/232) of patients in Model C aligned better with the actual outcomes. In contrast to the baseline model, the 3-month combined models exhibited a superior comprehensive improvement in actual outcome consistency, reaching 66.2%. Whether in the high or medium–low MMI cumulative dosage groups, the 3-month combined models showed enhanced concordance with actual outcomes compared to the baseline model.

For each patient, predicted risk probabilities were calculated from the baseline (Model A) and the early therapy (Models B and C) models. Model A categorized baseline predicted risks into three classes from low to high: Class I (< 52%), Class II (52%–71%), and Class III (≥ 71%). Simultaneously, early therapy predicted risks from Models B and C were categorized into four classes: Class I + (Model B: < 36%; Model C: < 21%), Class II + (Model B: 36–63%; Model C: 21–44%), Class III + (Model B: 63–83%; Model C: 44–63%), and Class IV + (Model B: ≥ 83%; Model C: ≥ 63%). Table 6 illustrates the distribution of varying classifications of risk among all three models and how the 3-month combined predictive model specifically influenced the refractory risk derived from the baseline model. For Class I patients with a baseline refractory predictive risk of < 52%, Model B (high cumulative dosage group, average ≥ 20 mg/day) elevated the risk for 10 out of 39 patients to 63%–83%, aligning closely with the actual refractory probability of 80%. The risk adjustment might lead them to lean towards RAI or surgical intervention at the early stage. Model C (medium–low cumulative dosage group, average < 20 mg/day) reduced the risk for 61 out of 144 patients to below 21%, instilling confidence in the continuation of ATD. Among Class II patients with a baseline refractory risk of 52%–71%, Model C reclassified the risk for 13 out of 58 patients to an average of 33%, aligning roughly with the actual risk. For Class III patients with a refractory risk of ≥ 71%, the number of individuals transitioning from Class III to Class I + or Class II + was minimal, indicating both high-dosage and medium–low-dosage groups maintaining a high risk of refractory GD.

**Table 6:** Distribution of risk classification for 3-month combined model and baseline model for refractory hyperthyroidism

	Model A			Total
	Class I (< 52%)^a^	Class II (52–71%)^a^	Class III (≥ 71%)^a^	
Model B (MMI ≥ 20 mg/day)				
Class I + (< 36%)^a^	15	8	0	23
	20% (3/15)^b^	38% (3/8)^b^	(–)	
Class II + (36–63%)^a^	14	11	10	35
	43% (6/14)^b^	55% (6/11)^b^	70% (7/10)^b^	
Class III + (63–83%)^a^	10	13	15	38
	80% (8/10)^b^	62% (8/13)^b^	67% (10/15)^b^	
Class IV + (≥ 83%)^a^	0	6	12	18
	(–)	100% (6/6)^b^	100% (12/12)^b^	
Total	39	38	37	114
Model C (MMI < 20 mg/day)				
Class I + (< 21%)^a^	61	0	0	51
	13% (8/61)^b^	(–)	(–)	
Class II + (21–44%)^a^	45	13	0	58
	29% (13/45)^b^	38% (5/13)^b^	(–)	
Class III + (44–63%)^a^	30	21	4	55
	60% (18/30)^b^	52% (11/21)^b^	50% (2/4)^b^	
Class IV + (≥ 63%)^a^	8	24	26	58
	50% (4/8)^b^	83% (20/24)^b^	85% (22/26)^b^	
Total	144	58	30	232

^a^Predicted risk based on Models A, B and C

^b^Numbers represent the actual number of refractory GD and the proportion in this group

### Sensitivity analysis through random forest models

**Fig. 7:**
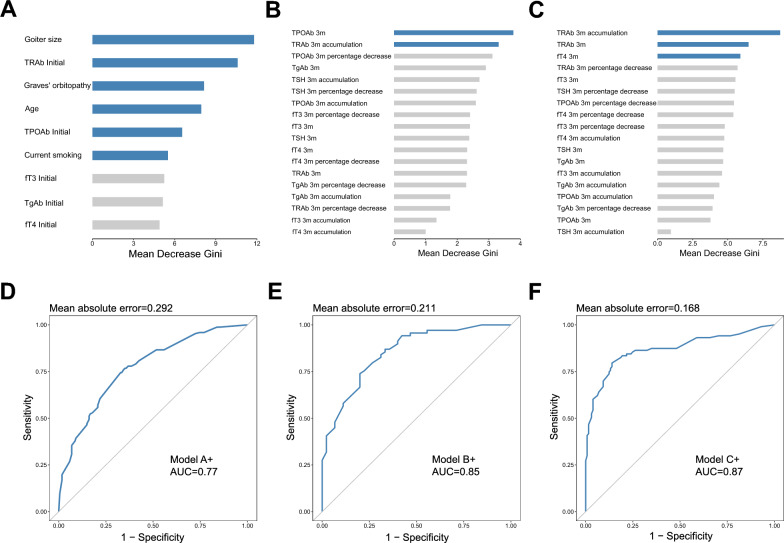
Random forest analysis results. **A** Variable importance ranking of Model A +. **B** Variable importance ranking of Model B +. **C** Variable importance ranking of Model C +. **D** *ROC* curve of Model A +. **E** *ROC* curve of Model B +. **F**. *ROC* curve of Model C +. *ROC* receiver operating characteristic curve, *AUC* area under the curve, *fT3* free triiodothyronine, *fT4* free thyroxine, *TSH* thyroid stimulating hormone, *TPOAb* thyroid peroxidase autoantibody, *TgAb* thyroglobulin autoantibody, *TRAb* thyroid stimulating hormone receptor autoantibody, *m* month. Model A +: baseline predictive model for total group (N = 346). Model B +: combined model of high cumulative MMI dosage group (≥ 1730 mg, average ≥ 20 mg/day, N = 114) at 3 months of therapy. Model C +: combined model of medium–low cumulative MMI dosage group (< 1730 mg, average < 20 mg/day, N = 232) at 3 months of therapy

As a supplementary method to interpret the complexity of the dataset and expand the scope of statistical models, a random forest analysis was conducted on the data. The process of feature selection determined a subset of variables most relevant to model building. With hyperthyroidism refractoriness as the dependent variable, age, current smoking, GO, goiter size, and initial fT3/fT4/TPOAb/TgAb/TRAb were analyzed. Based on the variable importance indicator *MDG*, six baseline variables were selected for all members of the analysis cohort, ranked in descending order of importance: goiter size, initial TRAb, GO, age, initial TPOAb, and current smoking (Fig. 7A). This formed the baseline validation model (Model A +) with an *AUC* of 0.77 and *MAE* of 0.292 (Fig. 7D). Model A and Model A + shared four model parameters: goiter size, initial TRAb, GO, and age. While Model A + exhibited a slightly stronger discriminative ability for refractory GD (*AUC* = 0.77 vs. 0.74), its calibration ability (*MAE* = 0.292 vs. 0.019) was inferior to Model A.

With hyperthyroidism refractoriness as the dependent variable, absolute values, percentage changes, and cumulative values of thyroid function and thyroid autoantibodies at three months of therapy were included. According to the *MDG*, 3-month therapy-related parameters were selected separately for the high cumulative and medium–low cumulative MMI dosage groups. The parameters of the high cumulative dosage group parameters were prioritized as follows: the absolute value of TPOAb at 3 months and the cumulative value of TRAb at 3 months (Fig. 7B). A total of 8 parameters were utilized to construct Model B + (*AUC* = 0.85, *MAE* = 0.211) (Fig. 7E), which included all parameters of Model A +. Model B and Model B + shared the same 3-month therapy-related parameters, in addition to the four baseline parameters. While Model B + demonstrates superior discriminative ability for refractory GD compared to Model B (*AUC* = 0.85 vs. 0.75), its calibration ability is poorer (*MAE* = 0.211 vs. 0.063). In the medium–low cumulative MMI dosage group, the importance ranking was the cumulative value of TRAb at 3 months, the absolute value of TRAb at 3 months, and the absolute value of fT4 at 3 months (Fig. 7C). Along with all parameters from Model A +, a total of 9 parameters were used to construct Model C + (*AUC* = 0.87, *MAE* = 0.168) (Fig. 7F). Model C and Model C + shared the same 3-month therapy-related parameters, in addition to the four baseline parameters. Model C + exhibits stronger discriminative ability for refractory GD compared to Model C (*AUC* = 0.87 vs. 0.80), but its calibration ability is lower (*MAE* = 0.168 vs. 0.028).

**Fig. 8:**
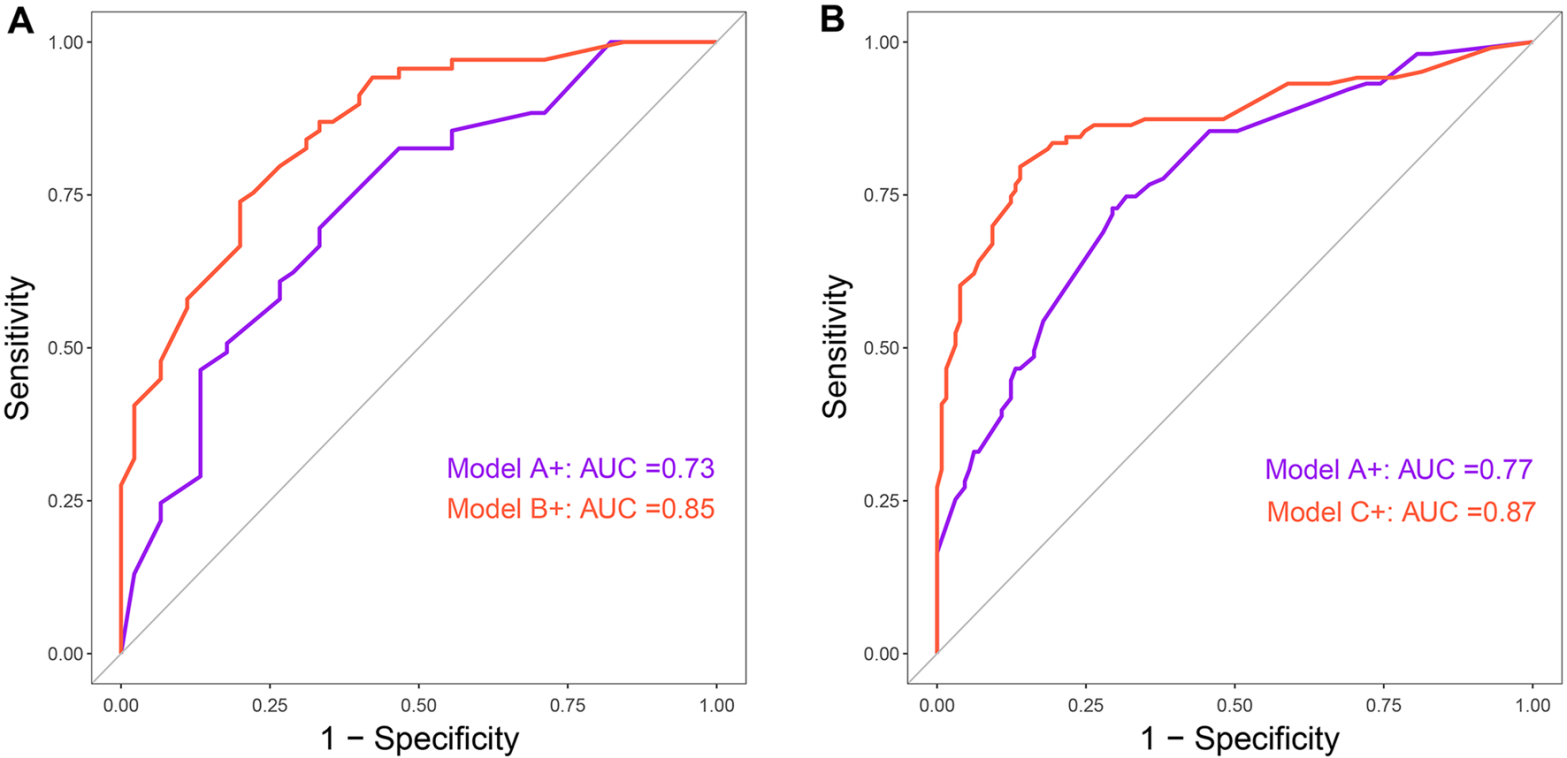
Comparison of *AUC* between different random forest models. **A** Model B + vs. Model A +. **B** Model C + vs. Model A +. Model A +: baseline predictive model for total group (N = 346). Model B +: combined model of high cumulative MMI dosage group (≥ 1730 mg, average ≥ 20 mg/day, N = 114) at 3 months of therapy. Model C +: combined model of medium–low cumulative MMI dosage group (< 1730 mg, average < 20 mg/day, N = 232) at 3 months of therapy

Finally, the discriminative abilities of the combined early-therapy and baseline random forest models were compared. In the random forest analysis, for the early high MMI cumulative dosage group, Model B + showed a higher *AUC* than Model A + (0.85 vs. 0.73) (Fig. 8A); for the early medium–low MMI cumulative dosage group, Model C + had a higher *AUC* than Model A + (0.87 vs. 0.77) (Fig. 8B). Consistent with traditional logistic prediction model results, the combined random forest models demonstrated superior discriminative ability in both high and medium–low MMI dosage groups compared to the baseline random forest model.

In summary, based on the present data, both random forest and logistic models performed well in predicting refractory GD. The combined models all demonstrated superior discriminative ability over the baseline models. While the overall discriminative ability of the random forest model was excellent, its calibration was weaker compared to the logistic regression model. The significant overlap in parameters between the two types of models further validated the importance and reliability of the variables selected by the logistic model. We ultimately chose the traditional logistic regression as the modeling method for refractory GD.

### Selection of antithyroid drugs dosage regimen after 3 months of therapy

**Fig. 9:**
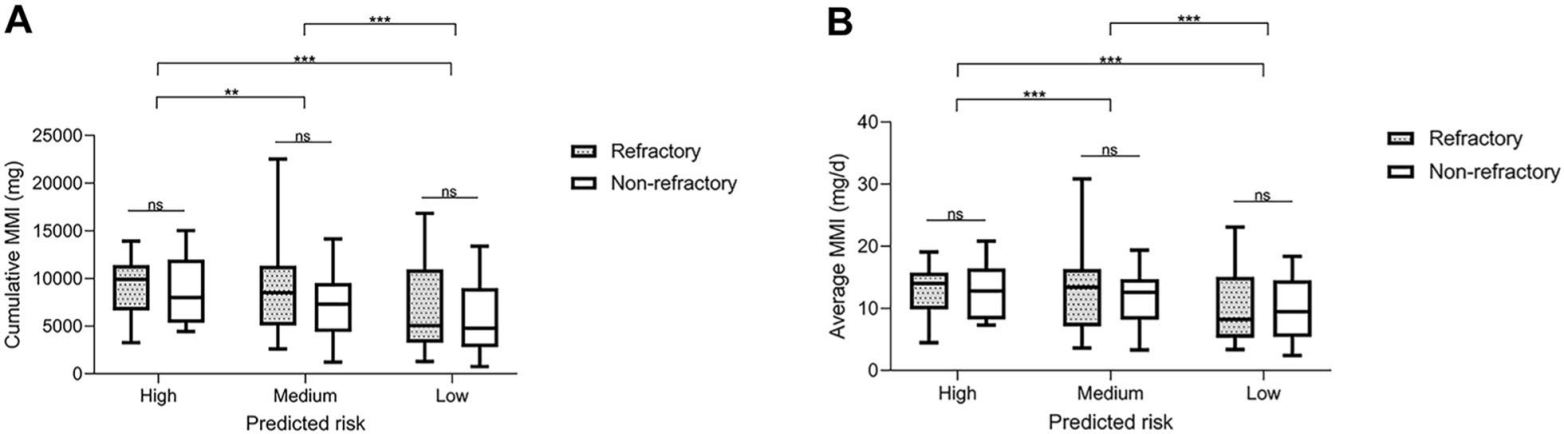
Analysis of refractory outcomes based on 2-year cumulative and daily average MMI dosage. **A** 2-year cumulative MMI dosage. **B** 2-year daily average MMI dosage. *MMI* methimazole. **P < 0.01; ***P < 0.001. High: High Predicted Risk Group (≥ 63%), Class III + and IV + in Model B and Class IV + in Model C. Medium: Medium Predicted Risk Group (36–63%), Class II + in Model B and Class III + in Model C. Low: Low Predicted Risk Group (< 44%), Class I + in Model B and Class I + and II + in Model C

After patients are assessed for risk during early therapy, a new question arises: Can conservative therapy effectively reduce the risk of refractory outcomes by adjusting medication dosage or extending treatment duration? Within our cohort, where the observation period was set at 2 years, we faced limitations in accurately assessing the influence of treatment duration, especially for prolonged therapies. Therefore, we only opted to analyze the 2-year MMI dosage to assess the impact of ATD dosing schemes on the prognosis of GD patients. Models B and C were examined, identifying merged groups as follows: High Predicted Risk Group (≥ 63%), Class III + and IV + in Model B and Class IV + in Model C; Medium Predicted Risk Group (36–63%), Class II + in Model B and Class III + in Model C; Low Predicted Risk Group (< 44%), Class I + in Model B and Class I + and II + in Model C. As shown in Fig. 9, a comparative analysis of 2-year cumulative and daily average MMI dosages among patients with different predicted risks revealed the following inter-group findings. Significant differences existed in the 2-year total MMI dosage among high, medium, and low predicted risk groups (*P* < 0.001). Post hoc tests indicated significant differences in pairwise comparisons between any two groups (high vs medium: *P* = 0.006; high vs low: *P* < 0.001; medium vs low: *P* < 0.001). Similarly, significant inter-group differences existed in the average daily MMI dosage among the high, medium, and low predicted risk groups (*P* < 0.001), with significant differences in pairwise comparisons between any two groups (high vs medium: *P* < 0.001; high vs low: *P* < 0.001; medium vs low: *P* < 0.001). The intra-group analysis demonstrated that in the high, medium, or low-risk groups, no significant difference existed in 2-year cumulative and daily average dosages between refractory and non-refractory patients (*P* > 0.05) (Fig. 9). These analyses suggested that patients in different refractory risk groups exhibited differences in 2-year cumulative MMI dosage and daily average dosage. However, no evidence suggested that adjusting MMI dosage can effectively improve the prognosis of refractory GD after early risk assessment.

## Discussion

GD, the most common cause of hyperthyroidism, is primarily treated with ATD in China, Japan, and Europe [30], while in the United States, the preferred treatment is RAI [31]. In our study cohort, the incidence of developing ATD-refractory hyperthyroidism in patients with newly diagnosed GD was 49.7%. Among these cases, one-third experienced recurrence after withdrawal, while two-thirds had persistent positive TRAb levels. The rates of TRAb persistence and recurrence after withdrawal are consistent with previous reports in Asian populations [32, 33]. However, our analysis cohort did not include patients who had switched to RAI or other medications. Patients in the cohort who switched to RAI and changed drugs cannot be ruled out from being affected by severe hyperthyroidism, drug insensitivity, or medication side effects [34]. In this case, they may also have ATD-refractory hyperthyroidism.

A considerable amount of clinical research exists on contributing factors to refractory hyperthyroidism, focused on the recurrence of hyperthyroidism [9, 21, 35, 36]. Poor treatment adherence is often an overlooked but crucial factor [8]. In this study, a relatively intensive follow-up schedule was implemented, with monthly follow-ups in the first six months and bi-monthly follow-ups thereafter, aiming to maximally enhance patient compliance. This study found that age, GO, goiter, initial fT3, fT4, and TRAb levels were all associated with refractory GD. Previous studies have indicated that younger patients have a lower response rate to antithyroid drugs and are more prone to relapse after withdrawal [20, 37]. In this study, patients under 36 years had a higher incidence of refractory GD. As a common complication of GD, GO was often encountered in our study cohort, primarily consisting of patients with mild to moderate GO. Those with severe symptoms or high clinical activity scores typically sought corticosteroid therapy or explore other treatment methods. To minimize interference with the analysis of MMI dosage, patients who had already undergone alternative treatments, which could cause interfere with the *OR* evaluation of GO, were excluded. The association between baseline goiter, GO, fT3, fT4, TRAb and the difficulty in achieving remission in GD has been confirmed by previous studies [20, 35, 38], consistent with our research findings. However, a study proposed that the association between goiter size and GD prognosis becomes insignificant after correcting for age and gender [39].

GD develops due to complex interactions among genetic, environmental, and endogenous factors. In clinical practice, the familial clustering of GD is common, primarily influenced by genetic factors, while the impact of regional or environmental factors on GD remains unclear [40]. Increasing evidence supports the relationship between genetic polymorphisms in GD patients and the remission rate after ATD therapy. Current research has identified polymorphisms in genes such as *CTLA-4*, *CD40*, *HLA* and *PTPN22* that may be associated with the prognosis of GD patients [40, 41]. Our study population included East Asian individuals from the Yangtze River Basin, and a limitation of the study was the lack of analysis of genetic factors and gene-related prognostic assessment in these patients.

Regarding the diet of GD patients, current research primarily focuses on iodine, selenium, and vitamin D. Both low and high levels of iodine may exacerbate thyroid autoimmunity, affecting the normal function of the thyroid gland. This could make GD more challenging to control or increase the likelihood of recurrence [42, 43]. Despite advising all patients in this study to follow a low-iodine diet during therapy, the iodine nutritional status of the patients was not monitored. Therefore, the impact of iodine intake on refractory GD cannot be determined. Additionally, selenium deficiency has been reported in GD patients, and selenium supplementation has been found to be beneficial for mild GO patients [44, 45]. Low vitamin D levels in GD may be associated with a higher relapse rate of hyperthyroidism after discontinuation of antithyroid drugs [46]. However, a recent multicenter randomized controlled trial by Rejnmark et al. suggested that vitamin D supplementation did not improve the treatment outcomes for GD patients with normal or insufficient vitamin D levels [47]. Dietary intervention or monitoring of vitamin D and selenium in GD patients were not implemented, hence the impact of vitamin D and selenium on refractory GD cannot be determined.

The predictive value of a single risk factor appears insufficient to forecast the outcomes of ATD therapy in patients. Therefore, at the initial diagnosis, a predictive model or clinical score based on multiple risk factors may be beneficial for guiding clinical decisions. Various models have been developed, including the Great score by Vos et al. [20] that incorporates age, fT4, thyrotropin binding inhibitory immunoglobulin (TBII), goiter size and its extended version, the Great + score that includes *HLA* polymorphisms and *PTPN22*. In addition, Masiello et al. [22] designed a clinical activity score—including factors such as goiter size, fT4, and GO—that provides valuable clinical guidance for predicting GD recurrence. However, existing predictive models related to GD have mainly focused on baseline characteristics, with limited research on re-evaluating risks after the initiation of therapy [20, 22, 48, 49]. Notably, research on predictive models for refractory GD is lacking, particularly regarding cases struggling to meet withdrawal criteria. Therefore, by defining the withdrawal criteria and limiting the treatment period, this study adopted a “progressive” study approach, examining risk factors associated with refractory GD at two time points: before therapy and at 3 months of therapy.

Regarding the changes in clinical characteristics at the 3-month mark of therapy, this study initially grouped patients based on the cumulative MMI dosage. Using an average of 20 mg MMI per day as a criterion, patients were divided into high and medium–low cumulative dosage groups. The thyroid function and autoantibodies of each group were then analyzed. The absolute values of TPOAb and the cumulative values of TRAb in the high dosage group at 3 months—as well as the absolute values of fT4, the percentage decrease in fT4, and the cumulative values of TRAb in the low dosage group at 3 months—were all robust predictors for future refractory GD during antithyroid drug therapy. Previous studies have confirmed that the decline in thyroid function and thyroid autoantibodies, especially TRAb or related subtypes, is highly correlated with the speed of normalization of thyroid function [50, 51]. The relationship between the changes in TPOAb and the prognosis of GD is debatable. Marcocci et al. [52] suggest that an increase in TPOAb levels is associated with an elevated risk of recurrence, while Stefanic et al. [53] hold the opposite view. Choi et al. [54] propose that this discrepancy may be linked to variations in the duration and ATD therapy protocols. Additionally, elevated levels of TPOAb may indicate a potential progress to Hashimoto's thyroiditis, ultimately leading to hypothyroidism. However, in our cohort, patients were not observed to transition from Graves’ hyperthyroidism to Hashimoto’s hypothyroidism. To the best of our knowledge, no other prospective study demonstrates the relationship between early treatment-related changes in thyroid function and the risk classification of refractory GD.

Based on multivariate analysis, a baseline (Model A) and combined early therapy (Models B and C) models were created. Patients were categorized into different groups with different refractory risks. Class III in the baseline model is close to the actual observed value. For these patients, subsequent evaluations at 3 months showed minimal changes, strongly suggesting that RAI might be more valuable than ATD therapy [18, 55]. For Class I and II patients, we found it necessary to regroup them based on the cumulative dosage at 3 months for a secondary risk assessment. Overall, the high cumulative dosage group exhibited a relatively higher risk. However, this finding does not imply a preference for lower-dosage MMI therapy because the medium–low-dosage group had relatively stringent clinical scoring criteria, as illustrated in nomogram plots (Figs. 4D and 5D). For example, the individual scores plotted on the nomogram at 3 months showed that the high-dosage group received a score of 17 points if the initial fT3 was ≥ 31.3 pmol/L, while the low-dosage group scored up to 35 points under the same condition. Finally, consistent with previous research [56–59], our analysis of the total MMI dosage over 2 years implied that the magnitude of MMI dosage cannot effectively alter the risk of refractory GD. Although our follow-up data are robust and prospective, a limitation of this study is the lack of randomization of therapy assignment within the cohort, making it challenging to eliminate the impact of subjective medication adjustments by doctors or patients. Our ongoing randomized study on ATD (unpublished) may address this problem.

The baseline model (Model A) rooted in baseline features is valuable during the initial diagnosis, assisting clinical physicians in identifying patients with a higher risk of refractory GD right from the start, especially those in Class III (refractory risk ≥ 71%). For such patients, ATD is not recommended as the primary treatment after diagnosis; instead, alternative treatments such as RAI or surgery are suggested. Patients in Class I and Class II, with lower baseline risks, can consider using ATD and have their risks reassessed in the treatment process. Compared with the baseline model (Model A), combined models (Models B and C) incorporate both baseline and 3-month therapy features, capturing the individualized evolution of GD under the influence of ATD. These models provide a dynamic risk assessment approach. The combined models readjust the predicted risks obtained at the baseline, enhancing the validity of the assessment. If the evaluation at the 3-month mark indicates a high predicted risk, such as Class III + and IV + in Model B and Class IV + in Model C (≥ 63%), it is recommended for such patients to discontinue ATD therapy to reduce unnecessary treatment duration or medical expenses. Physicians can promptly tailor treatment plans based on reevaluated risks for personalized care.

Worldwide research on refractory GD is ongoing, aiming to improve treatment outcomes and enhance the quality of life of patients. Some studies, including those conducted in China, have reported that, for the majority of GD patients, regular treatment over 5 years or longer results in long-term relief of hyperthyroidism, with no significant additional adverse effects observed in adults and children [6, 9]. However, the optimal duration of ATD therapy and factors influencing long-term prognosis remain uncertain [60, 61]. For patients unresponsive to ATD therapy, alternative treatments such as RAI or thyroidectomy are considered. Thyroidectomy is recommended for patients with severe GO or large goiter size, while RAI is suitable for elderly patients at high cardiovascular risk [62, 63]. Kim et al. suggest that the recurrence rate of RAI is higher in ATD-refractory GD patients compared to non-ATD-refractory GD patients [18]. This difference may be associated with thyroid enlargement and the impact of thyrotropin receptor antibodies (TBII), with no correlation found with the duration of previous ATD therapy [18]. For ATD-refractory GD patients who are unwilling to undergo thyroidectomy or RAI and prefer not to continue ATD, thyroid radiofrequency ablation may be a potential alternative treatment. However, patients with higher TRAb levels may still experience a relatively higher recurrence rate [64]. Additionally, for refractory GD patients with poor response to medications, especially those with persistent severe thyrotoxicosis, therapeutic plasma exchange may be considered as an option [13], but results from evidence-based medicine are insufficient to support this approach.

Predictive models based on baseline and early treatment characteristics have a certain degree of value in forecasting refractory GD. The strength of this study lies in the establishment of baseline and 3-month therapy assessment points, clear specifications for therapy duration and withdrawal criteria, and efforts to minimize interference of other medications with MMI. However, limitations include a relatively narrow definition of refractory GD, not accounting for recurrence risk and antibody changes in patients treated for over 2 years, and not considering patients who were forced to undergo alternative treatments due to uncontrolled thyroid function or severe complications. The predictive factors in this study’s model include cumulative values and percentage changes in thyroid function and antibody levels, which may limit its direct application in clinical assessments. A potential solution is to develop an assessment software for refractory GD, refining the model through iterative adjustments based on big data after expanding the study cohort [65, 66]. By automatically integrating and processing results, GD patients can be provided with guidance on personalized and precise therapy.

## Conclusions

The present study represents the first prospective study to evaluate the risk of ATD-refractory GD in Chinese population. By examining both baseline characteristics and early treatment responses, the research identifies significant risk factors—including younger age, GO, larger goiter size, and elevated levels of initial fT3, fT4, and TRAb at the time of diagnosis, as well as relevant indicators of ATD dosage, fT4, TPOAb, and TRAb at the 3-month therapy mark. The development of three predictive models, one based on baseline data (Model A) and two others incorporating baseline and early therapy information (Models B and C), demonstrates robust discriminative ability. Particularly noteworthy is the significant improvement achieved by combining baseline and 3-month therapy characteristics, enhancing the validity of predicting refractory GD outcomes compared to models relying solely on baseline information.

### Supplementary Information

**Additional file 1: **Refractory odds ratios for characteristics in the high cumulative dosage subgroup in univariable analyses.

**Additional file 2: **Refractory odds ratios for characteristics in the medium-low MMI cumulative dosage subgroup in univariable analyses.

## Data Availability

The datasets used and/or analysed during the current study are available from the corresponding author on reasonable request.

## Abbreviations

GD: Graves’ disease
ATD: Antithyroid drug
RAI: Radioactive iodine
TRAb: Thyrotropin receptor antibody
GO: Graves’ ophthalmopathy
fT3: Free triiodothyronine
fT4: Free thyroxine
TSH: Thyroid stimulating hormone
TPOAb: Thyroid peroxidase antibody
TgAb: Thyroglobulin antibody
MMI: Methimazole
ROC: Receiver operating characteristic
AUC: Area under the curve
HL: Hosmer–Lemeshow
MAE: Mean absolute error
MDG: Mean decrease Gini
OR: Odds ratio;
TBII: Thyrotropin receptor antibody;
TPE: Therapeutic plasma exchange
