# Supplementary material for: Enhanced predictive validity of integrative models for refractory hyperthyroidism considering baseline and early therapy characteristics: a prospective cohort study
Source: J Transl Med. 2024 Mar 29;22:318. doi: 10.1186/s12967-024-05129-3 (PMC10979605; doi:10.1186/s12967-024-05129-3)
Supplement: Supplementary file 1 — Additional file 1: Refractory odds ratios for characteristics in the high cumulative dosage subgroup in univariable analyses. [file 12967_2024_5129_MOESM1_ESM.docx]

**Additional file 1. Refractory odds ratios for characteristics in the high cumulative dosage subgroup in univariable analyses**

| Characteristics of Early Therapy  (High)^d^ | Univariate Analyses | | |
| --- | --- | --- | --- |
|  | Refractory, % (n/N) | *OR* (95% CI) | *P* Value |
| fT3 (3m)^a^ (pmol/L) |  |  |  |
| ≥8.0 | 71.1(32/45) | 2.1(1.0-4.7) | 0.064 |
| <8.0 | 53.6(37/69) | Reference |  |
| fT3 (3m percentage decrease)^b^ (%) |  |  |  |
| ≥59.0 | 56.1(55/98) | Reference |  |
| <59.0 | 87.5(14/16) | 5.5(1.2-25.4) | 0.030 |
| fT3 (3m accumulation)^c^ (day*pmol/L) |  |  |  |
| ≥588.5 | 62.6(67/107) | 4.2(0.8-22.6) | 0.096 |
| <588.5 | 28.6(2/7) | Reference |  |
| fT4 (3m)^a^ (pmol/L) |  |  |  |
| ≥21.2 | 66.7(34/51) | 1.6(0.7-3.4) | 0.229 |
| <21.2 | 55.6(35/63) | Reference |  |
| fT4 (3m percentage decrease)^b^ (%) |  |  |  |
| ≥61.0 | 58.1(54/93) | Reference |  |
| <61.0 | 71.4(15/21) | 1.8(0.6-5.1) | 0.262 |
| fT4 (3m accumulation)^c^ (day*pmol/L) |  |  |  |
| ≥1623.7 | 61.5(64/104) | 1.6(0.4-5.9) | 0.479 |
| <1623.7 | 50.0(5/10) | Reference |  |
| TSH (3m)^a^ (mIU/L) |  |  |  |
| ≥0.43 | 54.9(39/71) | Reference |  |
| <0.43 | 69.8(30/43) | 1.9(0.8-4.2) | 0.118 |
| TSH (3m percentage increase)^b^ (%) |  |  |  |
| ≥55403.3 | 54.2(26/48) | Reference |  |
| <55403.3 | 65.2(43/66) | 1.6(0.7-3.4) | 0.237 |
| TSH (3m accumulation)^c^ (day*mIU/L) |  |  |  |
| ≥115.8 | 58.9(33/56) | Reference |  |
| <115.8 | 62.1(36/58) | 1.1(0.5-2.4) | 0.732 |
| TPOAb (3m)^a^ (IU/mL) |  |  |  |
| ≥174.3 | 76.9(40/52) | 3.8(1.7-8.6) | 0.001 |
| <174.3 | 46.8(29/62) | Reference |  |
| TPOAb (3m percentage decrease)^b^ (%) |  |  |  |
| ≥11.2 | 55.9(38/68) | Reference |  |
| <11.2 | 67.4(31/46) | 1.6(0.7-3.6) | 0.219 |
| TPOAb (3m accumulation)^c^ (day* IU/mL) |  |  |  |
| ≥19734.8 | 76.6(36/47) | 3.4(1.5-7.7) | 0.004 |
| <19734.8 | 49.3(33/67) | Reference |  |
| TgAb (3m)^a^ (IU/mL) |  |  |  |
| ≥155.2 | 68.7(46/67) | 2.3(1.1-4.9) | 0.035 |
| <155.2 | 48.9(23/47) | Reference |  |
| TgAb (3m percentage decrease)^b^ (%) |  |  |  |
| ≥20.2 | 60.7(51/84) | 1.0(0.4-2.4) | 0.945 |
| <20.2 | 60.0(18/30) | Reference |  |
| TgAb (3m accumulation)^c^ (day* IU/mL) |  |  |  |
| ≥4849.4 | 65.1(54/83) | 2.0(0.9-4.6) | 0.108 |
| <4849.4 | 48.4(15/31) | Reference |  |
| TRAb (3m)^a^ (IU/L) |  |  |  |
| ≥4.9 | 65.3(47/72) | 1.7(0.8-3.7) | 0.176 |
| <4.9 | 52.4(22/42) | Reference |  |
| TRAb (3m percentage decrease)^b^ (%) |  |  |  |
| ≥12.7 | 61.3(57/93) | 1.2(0.5-3.1) | 0.726 |
| <12.7 | 57.1(12/21) | Reference |  |
| TRAb (3m accumulation)^c^ (day* IU/L) |  |  |  |
| ≥937.0 | 70.3(45/64) | 2.6(1.2-5.6) | 0.017 |
| <937.0 | 48.0(24/50) | Reference |  |

fT3, free triiodothyronine; fT4, free thyroxine; TSH, thyroid stimulating hormone; TPOAb, thyroid peroxidase autoantibody; TgAb, thyroglobulin autoantibody; TRAb, thyroid stimulating hormone receptor autoantibody; OR: odds ratio; CI: confidence interval; m, month.

a: Absolute serum levels at 3 months of MMI therapy.

b: Increase or decrease percentage of serum levels at 3 months of MMI therapy compared with the serum levels before therapy.

c: The area under the fitted curve of 0-3 months serum levels after the start of MMI therapy (the abscissa is the number of days, the ordinate is the fT3/fT4/TSH/TPOAb/TgAb/TRAb level).

d: 3-month high cumulative MMI dosage group (≥1730mg, average≥20mg/d, N=114).
