# Supplementary material for: Enhanced predictive validity of integrative models for refractory hyperthyroidism considering baseline and early therapy characteristics: a prospective cohort study
Source: J Transl Med. 2024 Mar 29;22:318. doi: 10.1186/s12967-024-05129-3 (PMC10979605; doi:10.1186/s12967-024-05129-3)
Supplement: Supplementary file 2 — Additional file 2: Refractory odds ratios for characteristics in the medium-low MMI cumulative dosage subgroup in univariable analyses. [file 12967_2024_5129_MOESM2_ESM.docx]

**Additional file 2. Refractory odds ratios for characteristics in the medium-low MMI cumulative dosage subgroup in univariable analyses**

| Characteristics during Early Therapy  (Medium-low)^d^ | Univariate Analyses | | |
| --- | --- | --- | --- |
|  | Refractory, % (n/N) | *OR* (95% CI) | *P* Value |
| fT3 (3m)^a^ (pmol/L) |  |  |  |
| ≥7.4 | 56.4 (44/78) | 2.1 (1.2-3.6) | 0.009 |
| <7.4 | 38.3 (59/154) | Reference |  |
| fT3 (3m percentage decrease)^b^ (%) |  |  |  |
| ≥64.5 | 40.0 (54/135) | Reference |  |
| <64.5 | 50.5 (49/97) | 1.5 (0.9-2.6) | 0.113 |
| fT3 (3m accumulation)^c^ (day*pmol/L) |  |  |  |
| ≥807.8 | 48.4 (74/153) | 1.6 (0.9-2.8) | 0.091 |
| <807.8 | 36.7 (29/79) | Reference |  |
| fT4 (3m)^a^ (pmol/L) |  |  |  |
| ≥21.0 | 57.5 (50/87) | 2.3 (1.4-4.0) | 0.002 |
| <21.0 | 36.6 (53/145) | Reference |  |
| fT4 (3m percentage decrease)^b^ (%) |  |  |  |
| ≥54.6 | 39.5 (62/157) | Reference |  |
| <54.6 | 54.7 (41/75) | 1.8 (1.1-3.2) | 0.030 |
| fT4 (3m accumulation)^c^ (day*pmol/L) |  |  |  |
| ≥3283.1 | 55.4 (36/65) | 1.9 (1.0-3.3) | 0.037 |
| <3283.1 | 40.1 (67/167) | Reference |  |
| TSH (3m)^a^ (mIU/L) |  |  |  |
| ≥0.66 | 40.3 (64/159) | Reference |  |
| <0.66 | 53.4 (39/73) | 1.7 (1.0-3.0) | 0.062 |
| TSH (3m percentage increase)^b^ (%) |  |  |  |
| ≥13032.4 | 39.8 (64/161) | Reference |  |
| <13032.4 | 54.9 (39/71) | 1.8 (1.1-3.2) | 0.033 |
| TSH (3m accumulation)^c^ (day*mIU/L) |  |  |  |
| ≥1095.5 | 25.0 (2/8) | Reference |  |
| <1095.5 | 45.1 (101/224) | 2.5 (0.5-12.5) | 0.276 |
| TPOAb (3m)^a^ (IU/mL) |  |  |  |
| ≥130.0 | 51.5 (50/97) | 1.6 (1.0-2.8) | 0.064 |
| <130.0 | 39.3 (53/135) | Reference |  |
| TPOAb (3m percentage decrease)^b^ (%) |  |  |  |
| ≥11.0 | 42.3 (58/137) | Reference |  |
| <11.0 | 47.4 (45/95) | 1.2 (0.7-2.1) | 0.448 |
| TPOAb (3m accumulation)^c^ (day* IU/mL) |  |  |  |
| ≥19039.5 | 52.0 (39/75) | 1.6 (0.9-2.7) | 0.108 |
| <19039.5 | 40.8 (64/157) | Reference |  |
| TgAb (3m)^a^ (IU/mL) |  |  |  |
| ≥92.2 | 49.0 (73/149) | 1.7 (1.0-2.9) | 0.060 |
| <92.2 | 36.1 (30/83) | Reference |  |
| TgAb (3m percentage decrease)^b^ (%) |  |  |  |
| ≥59.1 | 48.7 (19/39) | 1.2 (0.6-2.5) | 0.552 |
| <59.1 | 43.5 (84/193) | Reference |  |
| TgAb (3m accumulation)^c^ (day* IU/mL) |  |  |  |
| ≥26422.6 | 49.5 (50/101) | 1.4 (0.9-2.4) | 0.170 |
| <26422.6 | 40.5 (53/131) | Reference |  |
| TRAb (3m)^a^ (IU/L) |  |  |  |
| ≥6.9 | 56.3 (49/87) | 2.2 (1.3-3.7) | 0.005 |
| <6.9 | 37.2 (54/145) | Reference |  |
| TRAb (3m percentage decrease)^b^ (%) |  |  |  |
| ≥45.8 | 43.8 (42/96) | Reference |  |
| <45.8 | 44.9 (61/136) | 1.0 (0.6-1.8) | 0.868 |
| TRAb (3m accumulation)^c^ (day* IU/L) |  |  |  |
| ≥1051.5 | 62.0 (49/79) | 3.0 (1.7-5.3) | <0.001 |
| <1051.5 | 35.3 (54/153) | Reference |  |

fT3, free triiodothyronine; fT4, free thyroxine; TSH, thyroid stimulating hormone; TPOAb, thyroid peroxidase autoantibody; TgAb, thyroglobulin autoantibody; TRAb, thyroid stimulating hormone receptor autoantibody; OR: odds ratio; CI: confidence interval; m, month.

a: Absolute serum levels at 3 months of MMI therapy.

b: Increase or decrease percentage of serum levels at 3 months of MMI therapy compared with the serum levels before therapy.

c: The area under the fitted curve of 0-3 months serum levels after the start of MMI therapy (the abscissa is the number of days, the ordinate is the fT3/fT4/TSH/TPOAb/TgAb/TRAb level).

d: 3-month medium-low cumulative MMI dosage group (<1730mg, average<20mg/d, N=232).
